# Feasibility of automated insulin delivery guided by continuous glucose monitoring in preterm infants

**DOI:** 10.1136/archdischild-2019-316871

**Published:** 2020-04-15

**Authors:** Kathryn Beardsall, Lynn Thomson, Daniela Elleri, David B Dunger, Roman Hovorka

**Affiliations:** 1 Department of Paediatrics, University of Cambridge, Cambridge Biomedical Campus, Cambridge, Cambridgeshire, UK; 2 Neonatal Unit, Cambridge University Hospitals NHS Trust, Cambridge

**Keywords:** glucose, preterm, insulin, continuous glucose monitoring, closed loop

## Abstract

**Objective:**

Closed-loop systems have been used to optimise insulin delivery in children with diabetes, but they have not been tested in neonatal intensive care. Extremely preterm infants are prone to hyperglycaemia and hypoglycaemia; both of which have been associated with adverse outcomes. Insulin sensitivity is notoriously variable in these babies and glucose control is time-consuming, with management requiring frequent changes of dextrose-containing fluids and careful monitoring of insulin treatment. We aimed to evaluate the feasibility of closed-loop management of glucose control in these infants.

**Design and setting:**

Single-centre feasibility study with a randomised parallel design in a neonatal intensive care unit. Eligibility criteria included birth weight <1200 g and <48 hours of age. All infants had subcutaneous continuous glucose monitoring for the first week of life, with those in the intervention group receiving closed-loop insulin delivery in a prespecified window, between 48 and 72 hours of age during which time the primary outcome was percentage of time in target (sensor glucose 4–8 mmol/L).

**Results:**

The mean (SD) gestational age and birth weight of intervention and control study arms were 27.0 (2.4) weeks, 962 (164) g and 27.5 (2.8) weeks, 823 (282) g, respectively, and were not significantly different. The time in target was dramatically increased from median (IQR) 26% (6-64) with paper guidance to 91% (78-99) during closed loop (p<0.001). There were no serious adverse events and no difference in total insulin infused.

**Conclusions:**

Closed-loop glucose control based on subcutaneous glucose measurements appears feasible as a potential method of optimising glucose control in extremely preterm infants.

**What is already known on this topic?**

- Extremely preterm infants are prone to glucose dysregulation, which has been associated with adverse outcomes.
- Continuous glucose monitoring may help to identify hypoglycaemia and hyperglycaemia but targeting glucose levels remains challenging.
- Closed-loop systems have been used to optimise insulin delivery in children with diabetes, but they have not been tested in neonatal intensive care.

**What this study adds?**

- Closed-loop systems using subcutaneous glucose measurements to guide insulin delivery may improve glucose control in extremely preterm infants.
- Closed-loop systems could reduce the risk of hypoglycaemia associated with the use of insulin in this vulnerable population, but further studies are required.
- The use of insulin within closed-loop systems may help to optimise nutritional intake in these babies without increasing the risk of hypoglycaemia.

## Introduction

Preterm infants are at high risk of both hyperglycaemia and hypoglycaemia, related to deficits of insulin production and glycogen stores, with additive effects of parenteral nutrition, inotropic infusions and sepsis-related insulin resistance, resulting in extremely variable insulin sensitivity.^1 2^ Hyperglycaemia is observed in 80% of extremely preterm infants, and glucose variability is associated with increased mortality and morbidity.^2–6^ Moreover, at a practical level glucose control is difficult to achieve in an extremely preterm infant; often requiring multiple changes of intravenous infusions, insulin dosing, requiring extra attention of staff and therefore increased cost. The use of sliding scale insulin therapy is widespread but considered suboptimal as the desire to minimise blood sampling,^7^ combined with the extremely variable response to insulin,^1^ puts these babies at risk from hypoglycaemia.^8^ Babies are therefore often managed with a reduction in parenteral nutrition, and potentially inadequate nutritional support, when parenteral nutrition does not contribute as we might want to believe to hyperglycaemia and at a critical time of growth and development.^9 10^

Continuous glucose monitoring (CGM) has been used in neonatal care to identify hypoglycaemia and is increasingly being considered as a potential adjunct to support clinical management.^8 11–15^ However, the wide variation in individual insulin sensitivity and limited staff resources within intensive care make it challenging for the full potential of CGM to be realised.^14^ Adaptive computerised algorithms using blood glucose (BG) measurements have been evaluated in adults^16–18^ and neonates undergoing intensive care.^12 19^ The addition of frequent glucose levels obtained by CGM allows the development of closed-loop systems as recently investigated in adult intensive care patients.^20 21^ The present study hypothesised that closed-loop insulin delivery, based on subcutaneous CGM could be more effective in targeting glucose control in extremely preterm infants, compared with CGM with insulin therapy guided by a paper algorithm.

## Materials and methods

### Study design

Babies were recruited from the neonatal intensive care unit at University of Cambridge Addenbrooke’s Hospital NHS Trust. All parents of eligible babies admitted to the unit within 48 hours of birth were approached (if a member of the research team was available to do so). The study applied a randomised, open-label, parallel design, with babies randomised to CGM alone supported by a paper algorithm or to CGM with an additional intervention period of closed-loop CGM. Ethics Committee approval, and informed written parental consent were obtained prior to study procedures. Eligibility criteria included birth weight <1200 g and age <48 hours. Babies were excluded if they had a major congenital malformation, an underlying metabolic disorder or the mother’s pregnancy had been complicated by diabetes. Criteria were chosen based on previous data that identified these infants as at most risk from glucose dysregulation, while avoiding potential bias.^22^

All babies had real-time (CGM) inserted which remained in situ for up to 7 days. The paper guideline advised on the use of insulin or additional dextrose support. For a prespecified period of 24 hours, between 48 and 72 hours postbirth, a closed-loop system controlled glucose in babies during the closed-loop intervention, whereas babies in the control group continued to use CGM alongside the paper guideline. This window for closed-loop intervention was preselected as to be a standard time from birth in all babies, to allow time for informed parental consent to be obtained and as previous data (from masked CGM) had shown this to be the time when hyperglycaemia was most prevalent.^22^ Randomisation applied the minimisation methods using the Minim randomisation software,^23^ with stratification according to gestational age and birth weight.

### Common study procedures

Apart from glucose control over the prespecified period, all other aspects of care were identical between treatment groups. BG monitoring was undertaken on the point-of-care BG metre StatStrip  metre (Nova Biomedical, Waltham, Massachusetts, USA). Actrapid insulin (Novo Nordisk, Bagsværd, Denmark) in concentration of 25 U/kg in 50 mL of 0.9% saline was used in both treatment groups. For CGM, an Enlite sensor (Medtronic, Watford, UK) was inserted by hand into the lateral thigh of each baby, and linked to the Paradigm Veo (Medtronic) for display of the sensor glucose (SG) concentration. Outside the intervention period (48–72 hours), CGM data were used in combination with the paper algorithm, by the clinical team to guide glucose control in all babies. Nurses calibrated the CGM at least once every 12 hours with a BG measurement taken on the point-of-care BG metre.

### Paper algorithm

The paper algorithm for insulin delivery has previously been described (see online supplementary appendix 1).^14^ It provided guidance based on the absolute SG value as well as glucose trends. If SG levels were outside of the target range it advised to review the clinical care, dextrose infusion rate and to consider modifying insulin delivery, which could be instigated by the nurse at the cot side. The insulin and dextrose were delivered by Alaris pumps (CareFusion, San Diego, California, USA).

10.1136/archdischild-2019-316871.supp1 Supplementary data

### Closed-loop glucose control between 48 and 72 hours

The closed-loop system comprised (i) Enlite sensor, (ii) a laptop computer running a model predictive control algorithm and (iii) two Alaris syringe pumps. We used a control algorithm based on the model predictive control approach,^20^ optimised and tuned in silico using a computer simulation environment validated for glucose control in the critically ill,^24^ and aiming to keep SG between 4.0 and 8.0 mmol/L. The algorithm calculated insulin requirements or, at low glucose values, 20% dextrose infusion rates based on real-time SG values. A study nurse entered SG values into the laptop and modified insulin and dextrose pumps as directed by the control algorithm every 15 min. During the closed-loop intervention, actrapid insulin in concentration of 5 U/kg in 50 mL of 0.9% saline was used to allow for finer dose titration in accordance with the algorithm. The algorithm calculations used a compartment model of glucose kinetics^25^ describing the effect of insulin on SG excursions. The algorithm was initialised using a baby’s weight and adapted itself to a particular baby by updating two model parameters—a rapidly changing glucose flux correcting for errors in model-based predictions and a slowly changing estimate of an insulin rate to maintain normoglycaemia. The individualised model forecasted plasma glucose excursions over a 1.5 hour prediction horizon when calculating the insulin rate and a 30–40 min horizon when calculating the dextrose rate. Information about enteral or parenteral nutrition was not provided to the algorithm. A reference BG value was used every 6 hours for calibration of glucose sensor. If sensor readings were not available due to sensor failure or loss of data capture, then hourly BG levels were used to inform the algorithm for up to 4 hours. At this time, the algorithm continued to provide advice every 15 min.

### Assessments and data collection

Demographic and clinical characteristics were collected with antenatal variables defined as: antenatal steroids as having received at least one dose prior to delivery, prolonged rupture of membranes as rupture >24 hours prior to delivery, hypertension and chorioamnionitis were based on clinical diagnoses recorded in the maternal medical file. All BG measurements, insulin administration, type and volume of enteral and parenteral nutrition and additional intravenous glucose administration were recorded from the time of randomisation to the end of CGM.

### Statistical analysis

The outcome measures and the statistical analysis plan were agreed in advance. Primary outcome being comparison of time SG in target glucose range 4.0–8.0 mmol/L between study arms. Secondary outcomes were time spent with SG levels between 2.6 and 10.0 mmol/L, prevalence of hyperglycaemia (per cent time SG >10.0 mmol/L), mean and SD of SG. Safety end points included frequency of hypoglycaemia (any BG <2.6 mmol/L) and other adverse events. Sample size was selected to reflect this study as a pilot, to ensure we had comparable control data, while minimising patient exposure. Formal power calculations were not performed, and all analyses are based on intention to treat. Unpaired t-test was used to compare normally distributed variables. Non-normally distributed variables were compared using Mann-Whitney U test. Calculations were carried out using SPSS V.23 (IBM Software, Hampshire, UK). Values are given as mean, SD or median (IQR). P value <0.05 was considered to be statistically significant.

## Results

Twenty-one babies were randomly assigned, between study arms, but one baby in the control group died within 48 hours of birth, so was excluded from the analyses. Baseline characteristics of the two groups (n=20) were similar (table 1). There were no significant problems with sensor insertion or removal, with only one baby (control group) having a sensor replaced as there was no connection to the monitor. All 20 babies remained in the study throughout the intervention period from 48 to 72 hours with comparable amount of SG data available for analyses in each group (median (IQR) for both study groups 24 hours (23.75, 24.00)). Control algorithm directed insulin therapy was followed, at all times, during the prespecified 48–72 hours period. The maximum period of sensor signal loss during the closed loop was 3.5 hours, during which hourly BG values were used by the control algorithm. For the remaining study, there was minimal loss of data: one baby in the ‘control’ group was transferred out of the unit on study day 5, and one baby in the closed-loop arm died on study day 6, so no further data could be collected. Therefore, the mean (SD) length of glucose data collected in each study arm were 137 (16.4) hours and 136 (8.7) hours for CGM and CGM plus closed loop, respectively.

**Table 1:** Baseline demographic data

	Closed loop (n=10)	Control (n=10)
Gestational age at birth (week)	27.0 (2.4)	27.5 (2.8)
Birth weight (g)	962 (164)	823 (282)
Sex (male:female)	5:5	5:5
Antenatal variables		
Antenatal steroids	10 (100%)	9 (90%)
Maternal smoking	1 (10%)	2 (20%)
Chorioamnionitis	2 (20%)	3 (30%)
PROM	4 (40%)	4 (40%)
Hypertension	1 (10%)	1 (10%)

Data are presented as mean (SD).

PROM, prolonged rupture of membranes (>24 hours).

### Glucose control, insulin and dextrose administration between 48 and 72 hours

There was no difference in the baseline mean SG at 48 hours between study groups (table 2). During the intervention period, the median (IQR) time spent in the target range (SG 4.0–8.0 mmol/L) was significantly higher in babies in the closed-loop group 91% (78, 99) compared with controls 26% (6-64); p<0.001—equivalent to 22 (19-24) hours in the ‘closed loop’ versus 6 (1-15) hours in the control group. Similarly, the time spent in the wider target range 2.6–10.0 mmol/L was higher in the closed-loop group: median 100% compared with control group, median 84%. This was due to the smaller per cent of time with SG values >10 mmol/L with median 16% in the control group compared with median 0% in the closed-loop group. There was no difference in the time spent with SG levels <2.6 mmol/L. Lower SG was observed in the closed-loop group median (IQR) 6.2 (6.1-7.1) mmol/L compared with the control group 8.6 (7.4-11.1) mmol/L (p=0.002). Glucose variability as measured by the SD of SG was similar (p=0.604).

**Table 2:** Comparison of glucose control, insulin delivery and nutritional intake during the intervention period (48–72 hours postbirth)

	Closed loop (n=10)	Control (n=10)	P value
Time spent with sensor glucose level (%)			
4.0–8.0 mmol/L*	91 (78-99)	26 (6-64)	<0.001
2.6–10 mmol/L	100 (94-100)	84 (46-98)	0.133
>10.0 mmol/L	0 (0-6)	16 (2-54)	0.113
<2.6 mmol/L	0.0 (0.0-0.0)	0.0 (0.0-0.0)	0.720
Baseline sensor glucose (mmol/L)	7.9 (6.9-11.5)	8.2 (7.0-12.4)	0.182
Mean sensor glucose (mmol/L)	6.2 (6.1-7.1)	8.6 (7.4-11.1)	0.002
SD of sensor glucose (mmol/L)	1.0 (0.8-1.9)	1.3 (0.9-2.5)	0.604
Episodes of blood glucose <2.6 mmol/L†	1	0	1.000
Insulin (U/kg/hour)	0.04 (0.03-0.07)	0.02 (0.00-0.11)	0.400
Nutritional intake			
Dextrose (mg/kg/min)	8.4 (7.2-10.3)	8.5 (4.2-10.6)	0.604
Protein (g/kg/day)	3.2 (2.5-4.1)	3.5 (1.6-4.1)	1.000
Lipid (g/kg/day)	1.8 (1.0-1.8)	1.4 (0.9-2.2)	0.905
Trophic feeds	4	4	1.000

Data are presented as median (IQR).

*Primary end point.

†Present at start of closed-loop study period prior to computer algorithm advice being initiated.

**Figure 1:**
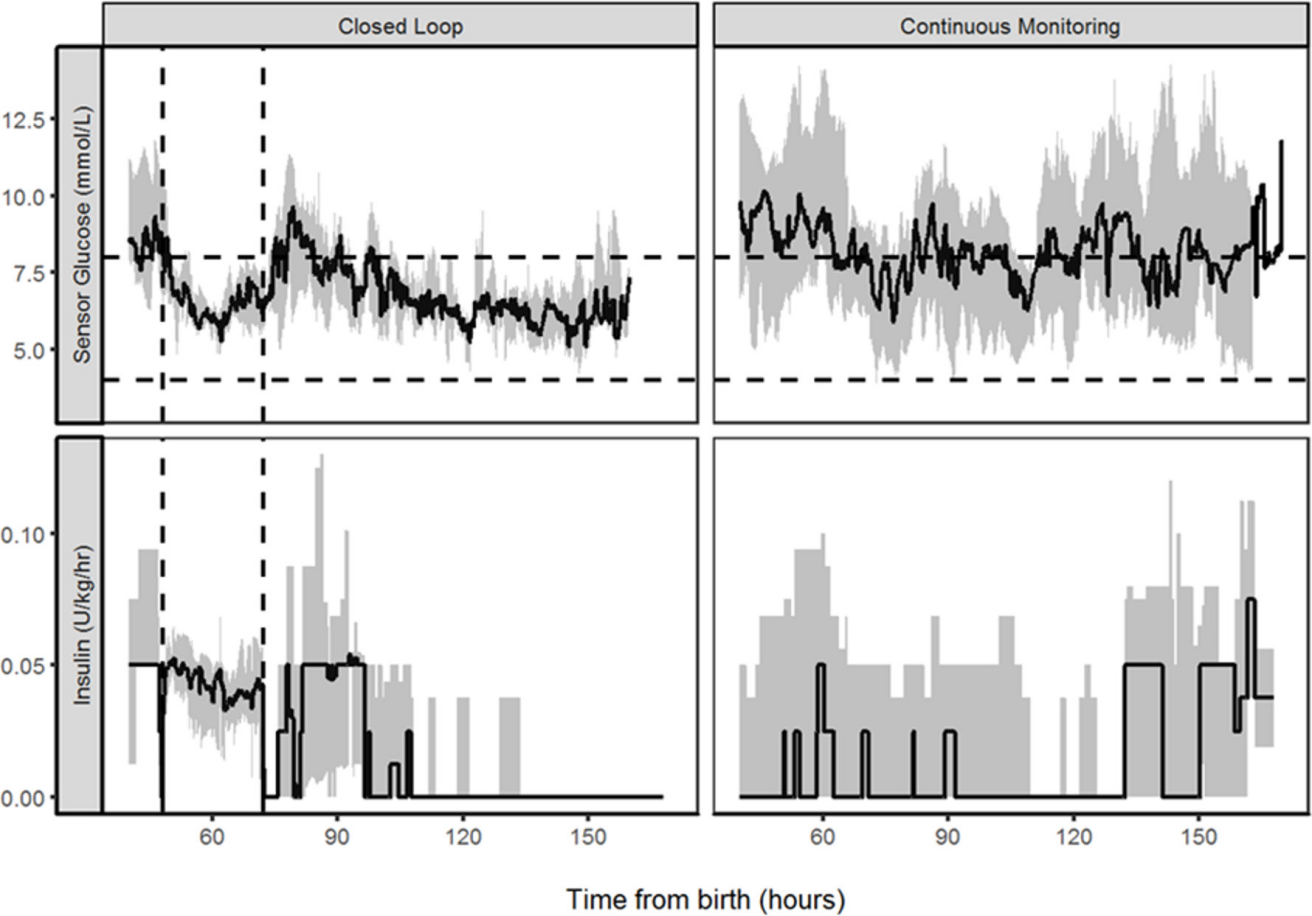
Glucose control and insulin delivery median (IQR) of sensor glucose and insulin infused in babies randomised to closed-loop management or continuous glucose monitoring with paper algorithm (control). The closed-loop intervention period is denoted by the vertical lines, and the target glucose range 4.0–8.0 mmol/L is denoted by horizontal lines.

The summative glucose profiles and insulin infused are provided in figure 1. Nine out of the 10 babies in the closed-loop group had received insulin prior to the 24 hours intervention with the one remaining baby being started on insulin during the 24 hours closed loop. This compared with four babies in the control arm having received insulin prior to, and eight babies receiving insulin during the intervention period. Four babies in the closed-loop study arm received additional 20% dextrose for short periods during the intervention period (up to 3.5 hours). The mean infusion rate in these babies ranged from 0.13 to 0.53 mL/kg/hour. The highest rate being infused in a baby who was hypoglycaemia prior to the start of the closed-loop intervention. There was no statistical difference in the total amount of insulin infused or nutritional intake between study groups during the 24 hours intervention period.

### Glucose control, insulin and dextrose administration between 72 and 160 hours

In the postintervention period, after 72 hours, there was a trend of increased time in both glucose target ranges (4.0–8.0 and 2.6–10.0 mmol/L) in the closed-loop group compared with the control group (table 3), but these differences did not reach statistical significance.

**Table 3:** Comparison of glucose control, insulin delivery and nutritional intake during the postintervention period (72–160 hours postbirth)

	Closed loop (n=10)	Control (n=10)	P value
Time spent with sensor glucose level (%)			
4.0–8.0 mmol/L	64 (39-90)	42 (30-55)	0.053
2.6–10 mmol/L	95 (79-97)	78 (61-97)	0.243
>10 mmol/L	3 (1-21)	22 (3-36)	0.156
<2.6 mmol/L	0.0 (0.0-0.8)	0.0 (0.0-0.2)	0.720
Mean sensor glucose (mmol/L)	7.0 (6.8-8.5)	8.3 (7.3-9.2)	0.182
SD of sensor glucose (mmol/L)	1.7 (1.5-2.1)	1.7 (1.3-2.8)	0.780
Episodes of blood glucose <2.6 mmol/L	1	0	1.000
Insulin (U/kg/hour)	0.02 (0.00-0.04)	0.03 (0.00-0.05)	0.356
Nutritional intake			
Dextrose (mg/kg/min)	9.4 (7.0-10.6)	8.7 (5.4-10.9)	0.549
Protein (g/kg/day)	3.7 (2.7-4.3)	3.8 (2.9-4.4)	0.968
Lipid (g/kg/day)	2.0 (1.5-2.9)	1.8 (1.2-2.8)	0.611
Oral milk intake (mL/kg/day)	4.4 (3.5-11.5)	5.0 (0.5-13.0)	0.720

Data are presented as median (IQR).

### Nutrition and clinical care

All babies received parenteral and enteral nutrition according to the standard local neonatal unit protocol. During the closed-loop intervention period between 48 and 72 hours, four babies in each study group were receiving minimal amounts of trophic feeds. In the postintervention period, after 72 hours, there was no difference in the volumes of milk received between the two study groups (table 3). None of the babies received hydrocortisone, but seven babies in the control arm and two in the intervention arm received inotropes during the first week. During the intervention period, the median (IQR) number of BG values taken per day was 5.50 (4.75-6.56) in the control group compared with 6.25 (5.13-6.5) in the intervention group and over the whole study period 5.14 (4.29-5.43) in the control compared with 5.29 (4.64-5.80) in the intervention group.

### Safety

There were no reported concerns about the sensor site in terms of inflammation or infection either during the study or after removal. In the closed-loop study group, there were two babies who had documented episodes of hypoglycaemia with BG <2.6 mmol/L. One episode occurred when checking the baseline BG prior to the onset of closed loop, at this time maintenance fluids were being changed, but no insulin was being infused. The second episode was on day 6, again associated with a change of maintenance fluids. There were a further two babies who had periods (after the 72 hours closed loop) when SG fell to below 2.6 mmol/L but the BG at this time was documented above 2.6 mmol/L. In the control study group, no babies had a documented BG value <2.6 mmol/L. One baby in the control group had an episode lasting 205 min when the SG fell to <2.6 mmol/L (BG was not checked at this time). None of the babies were on insulin, and there was no clinical evidence of hypoglycaemia in these babies during any of these episodes.

## Discussion

This study is the first to show that a closed-loop system using subcutaneous glucose measurements to guide insulin delivery may improve glucose control in extremely preterm infants. This new approach could represent a step-change in care, providing greater safety and tighter control while minimising staff time at bedside and changes in fluid/insulin treatment. Compared with CGM alone, closed-loop intervention increased the time  when SG was in the target range of 4 to 8 mmol/L threefold. In the high-intensity and high-cost setting of neonatal intensive care, this preliminary data support further development of closed-loop systems, with real-time glucose-responsive insulin and dextrose delivery to support the care of these babies.

Reflecting the current controversy regarding optimal targets for glucose control in neonatal intensive care, we adopted a moderate glucose target range between 4.0 and 8.0 mmol/L rather than the tight glycaemic regimen of Leuven and NICE-SUGAR (Normoglycaemia in Intensive Care Evaluation and Survival Using Glucose Algorithm Regulation) studies.^26 27^ These moderate target ranges represent physiological in utero levels,^28^ and the upper threshold reflects a postnatal glucose level which has been associated with increased mortality and morbidity in preterm infants.^29^ However, the optimal target for glucose levels in these babies remains to be determined and this study does not resolve this long-standing debate in the field. It rather shows how an automated system can be used to achieve control to a given target range.

Different strategies are currently used to target glucose control in the preterm infant, each with different risks and benefits. A reduction in dextrose load risks compromised nutritional intake, while insulin therapy can lead to hypoglycaemia. The level of insulin use within this study is likely to reflect both the extreme prematurity of the babies recruited and the current practice on our unit, which aims to ‘optimise’ nutritional delivery. In this study, there were no episodes of hypoglycaemia related to advice from the closed-loop algorithm, but the use of the CGM highlighted clinically silent episodes of hypoglycaemia in both study arms, independent of insulin use. The prevalence of hypoglycaemia was comparable to that reported by others in this population (27%),^30^ and to that reported using masked CGM which highlights prolonged periods of clinically silent hypoglycaemia.^22^

The CGM were calibrated using the StatStrip metre that is validated for use in neonates and has the Food and Drug Administration approval for use in intensive care.^31^ These metres were used, as they were the standard of care for clinical management within the neonatal unit. Although there remains controversy regarding the clinical significance of clinically silent episodes of hypoglycaemia detected on CGM, there is recent evidence of an association between these episodes with substantially increased risk of impaired executive function and visual motor difficulty at 4.5 years.^32^

The frequency of BG sampling in preterm infants is typically much lower than in adults and children in intensive care and therefore the use of CGM has the potential to have an even greater impact. Differences in frequency of BG monitoring may have had an impact on level of glucose control. However, within the control arm babies had >4 BG values taken every 24 hours in the first 3 days of life. Of note, the strength of insulin in the intervention period was more dilute than in the comparative control group to allow for more frequent changes in dose titration. Despite randomisation, there were a larger number of babies in the control arm who received inotropes during the first week, and inotropic drugs will reduce insulin secretion and insulin sensitivity. However, given the pattern of improved glucose control during the intervention we do not think these differences could account for the differences in glucose control that were observed.

Previous studies have explored the potential for the use of CGM to guide changes in dextrose delivery,^12^ these studies though remained dependent on staff responding to trends or alarms in SG before intervening.^12^ This contrasts with the present study in which the targeting of glucose control is proactively driven by the closed-loop algorithm, which was responding to frequently sampled SG data. This study is unique in exploring a control approach belonging to the family of model predictive control algorithms and optimised on a validated computer simulation environment^24^ prior to study commencement to ensure a favourable outcome.

The strengths of our study are the randomised controlled study design and comparability of the study groups and nutritional intakes. The study limitations include a small sample size, where despite randomisation there can remain base line differences between study groups. Although the study groups appear comparable, more babies in the control group compared with the intervention group were on inotropes, and more babies in the intervention arm were on insulin at the start of study intervention. In such a design, it was not possible to blind the clinicians to the intervention. The study duration was short, and as it is known that insulin sensitivity varies over time from birth, how the algorithm works over longer periods would be important to explore. As a single-centre study design, where nutritional policies and practices will vary in comparison to other units it would be important to test the algorithm in different units with different nutritional strategies to test generalisability. The algorithm was not designed to alter nutritional delivery, as this was predetermined by the clinical team; with the intervention designed as a simple adjunct to support targeting glucose control, alongside the clinical plan for nutritional support. Alternative designs might consider a combined approach to guide nutritional intake alongside insulin treatment. Further studies are required to explore the impact of a fully automated system with infusion pumps providing insulin and 20% dextrose under fully automated computer control.

This is the first randomised study to evaluate the feasibility of a closed-loop control, based on CGM in preterm infants to guide insulin delivery to support glucose control. This closed-loop system appears a potential adjunct for targeting glucose control in preterm infants requiring intensive care and warrants further study.
